# Responsibility of the Army Surgeon

**Published:** 1863-10

**Authors:** 


### RESPONSIBILITY OF THE ARMY SURGEON.

When suffering or calamity befalls or swift destruction overtakes any one, the first inquiry and most natural is as to its cause. "Some one has blundered," or worse, and the disposition is frequently evinced to censure others than the sufferers though entirely innocent. A steam boiler *may* explode and the engineer be entirely guiltless of the consequences; and so many a man languishes and dies through no fault of his medical attendant. The disposition to uncharitable animadversion is even more frequent and unreasonable toward the medical men connected with military hospitals than toward men of the same ability and professional devotion in private practice. A brief *resume* of the duties and privileges of the medical corps will be found elsewhere in this number, and to it we beg to refer the reader for an exposition of the status of the medical officer in the army. We do not now propose to discuss the question as to whether or no his position or privileges are exactly what the interests of the army and humanity require they should be; our present aim is to correct a misunderstanding which is quite general and mischievous.

The surgeon has the immediate care of the sick and wounded of our armies. Over these, when once in his hands, his control is complete; but as to the matters which relate to the health of the command, his power is merely *advisory*. He cannot command the abatement of a perilous nuisance, neither can he order the removal of a camp from an unhealthy to a healthy site. He cannot compel the digging of a sink, the draining of a cesspool, the proper ventilation of quarters, cleanliness of camp, clothing or person, or exercise authoritative supervision over the cookery, habits, or exercise of the men. Orders in all these cases must be issued by the military head. These facts are mentioned, not as indicating any adverse opinion as to their propriety, but as facts which should be more generally understood. There is frequently—perhaps generally—that perfect harmony of purpose and co-operative action between the medical and military departments, which devolves the hygienic management of the command as entirely upon the surgeon as the administrative and tactical in the hands of the commanding officer; but the exceptions to this rule are, unfortunately, by no means rare. In the instances in which there is not this strict and hearty concert of action, the health of the command may suffer severely from preventable disease through no fault of the surgeon.

Necessity is the higher law in military campaigning, and frequently demands the infraction of well-recognized sanitary measures. Exhausting marches, innutritious food, short rations, insufficient clothing, camping grounds reeking with poisonous exhalations, are often accepted as risks which are indispensable to the accomplishment of the objects of the campaign. Whilst much may be done by way of mitigating the effects of these and other adverse conditions, they must frequently be encountered as constituting portions of the extra hazard inseparably connected with military life.

Perhaps the surgeon is more hampered in his efforts to serve his command by the absence of independent transportation than by any other single regulation. Paragraph 1,103, Revised Army Regulations, provides that "the baggage trains, ambulances, and all the means of transport continue in charge of the proper officers of the Quartermaster's Department, under the control of the commanding officers." The surgeon is the only official representative of those who, whether from sickness or wounds, are unable to fight. During an active campaign, this class is an embarrassing and vexatious hindrance and the difficulty encountered by the surgeon in taking proper care of these unfortunates without a real or apparent weakening of the available force, is often very great. Before the days of the great Pare, the father of French and military surgery, the wounded were frequently abandoned to the uncertain sympathies and succor of the inhabitants of the country where the battle had been fought. Pare organized a system of military hospitals which moved with the army, and originated the idea of an ambulance operating in the immediate rear of the scene of action. Great improvements have been adopted since then in military surgery, in the appliances and management of field and general hospitals, and in the structure of the ambulance; but now, as then, the medical officer is entirely dependent upon the disposition and action of non-medical men for the possession of the necessary facilities for caring for those whom the fortunes or misfortunes of war have thrown upon his hands. One of the problems most carefully pondered by the conscientious surgeons in this war has been how they could best utilize their *material* for the good of their charge. Many efforts have been made in the various armies of the Union to properly systematize the ambulance department, but thus far without anything approaching satisfactoriness, mainly because Congress has failed to legalize and organize an ambulance corps. The same holds true in regard to the difficulty frequently experienced in procuring their medical supplies. On various occasions the medical transportation has been so effectually locked up and lost in the chaos of baggage trains, that the surgeons have been entirely destitute of their regular supplies for days after an engagement—depending exclusively upon the resources of the neighborhood, and those which had, somehow or other, been got through by the Sanitary Commission. To mention no lesser instances, this was especially true of the great battles of South Mountain, Antietam and Perryville.

Having shown some of the impediments in the way of the usefulness of the army surgeons, it only remains for us to say that, in view of all the difficulties—necessary and otherwise—the medical staff has accomplished all that could be reasonably expected of it. An opportunity unusually favorable for forming a correct estimate of the moral and professional status of the medical corps in the Department of the South, in the Army of the Potomac, and in that before Vicksburg, has led us to believe that as a class those comprising it have been grossly misjudged and misrepresented. Exceptions there are, but we do not hesitate to say that the most hard-working, self-denying earnest, and conscientious officers in the army are its surgeons. True, "there are those among them so ignorant, and others so depraved, that they are a curse to the service, *opprobria* to the professin, and a disgrace to those by whom they were commissioned, yet such are comparatively few; most are laborious, faithful and meritorious. The greater part have passed a rigid examination before a competent medical board, by whom they were declared well qualified for their duties, and have since, by their services in the field, vindicated the propriety of their selection from the great number of candidates for the places which they hold. During the past year and a half all the surgeons have been overworked; few have escaped disease contracted in the discharge of their duties; many have gone home permanently broken, and not a few have been martyrs to their faithfulness."—*Sanitary Reporter.*

